# Precision Medicine in Periodontics: A Literature Review

**DOI:** 10.7759/cureus.68952

**Published:** 2024-09-08

**Authors:** Mrunalini Gundelly, Santosh V Pusuluri, Rekha R Koduganti, Manasa Ambati, Sneha Chiluveru, Meenakshi Chandaka

**Affiliations:** 1 Periodontics, Panineeya Mahavidyalaya Institute of Dental Sciences and Research Centre, Hyderabad, IND

**Keywords:** biomarkers, gcf sample, periodontitis, precision in diagnosis, saliva sample, traditional diagnostic techniques

## Abstract

Periodontitis, a widespread health issue, requires effective prevention and management strategies due to its increasing prevalence and detrimental social consequences. The chronic inflammation associated with periodontitis also exacerbates systemic conditions, emphasizing the need for advanced approaches in addressing this public health concern. The traditional methods of periodontal diagnosis, which primarily rely on clinical indicators such as pocket depth, clinical attachment loss, mobility, and radiographic measurements of alveolar bone loss, have limitations in guiding therapy due to the intricate and multifaceted nature of periodontal diseases. Precision periodontics is the amalgamation of genomics, bioinformatics, and advanced technology, mainly biomarkers reflecting a precise patient-centered treatment. However, implementing this approach in periodontology is new due to the lack of validated periodontal biomarkers for diagnostic use. This article explores the foundations of personalized therapy in periodontal diagnosis. It discusses the current state and prospects of periodontal biomarkers as a crucial step toward realizing a precision approach in periodontal practice.

## Introduction and background

Periodontitis poses a significant public health concern, as evidenced by its ranking as the 11th most prevalent condition worldwide according to the Global Burden of Disease Study (2016) [1]. The incidence of severe disease ranges from 20% to 50% globally and is a major contributor to tooth loss, which can negatively impact masticatory function, aesthetics, self-confidence, and overall quality of life [2]. As a result, a concerted effort has been made to enhance preventative and therapeutic strategies for periodontal care to minimize diagnostic errors, improve outcomes, and mitigate the economic burden associated with periodontal disease. Plaque is a harbinger of periodontitis, and it is imperative that the plaque biofilm has to be disrupted for periodontal treatment to be successful. Periodontitis is understood to be an interaction between the dysbiotic microflora thriving in the plaque biofilm and dysregulated inflammation [3]. As the disease is multifactorial, the use of biomarkers could be more beneficial when compared to the traditional clinical and radiological diagnostic measures; this enables highly reliable prediction of periodontal disease susceptibility, early diagnosis, prognosis, and planning of the most effective and safe treatment strategy to meet individual patient needs [4]. In vitro diagnostics (IVD) is employed in over 60% of all medical decisions today, providing a more effective method of health care delivery. Biomarkers have been introduced in the new classification of periodontal and peri-implant conditions (2017) as a preliminary step toward implementing precision medicine concepts in periodontology. This development highlights a leap in the field of diagnostics [5]. Precision medicine is a tailored therapeutic approach relying on an individual's unique genetic, biomarker, epigenetic, phenotypic, and socioeconomic or psychosocial traits to deliver personalized healthcare. While this concept has caught up in various medical specialties, its implementation in periodontology has been hindered by the lack of validated biomarkers for diagnostic use.

## Review

Restrictions of conventional diagnostic methods

The traditional diagnostic methods for periodontal diseases, such as clinical and radiographic assessments of attachment loss, are subject to inherent inaccuracies. Additionally, individual susceptibility to periodontitis can vary genetically and temporally, presenting a diagnostic challenge. These conventional techniques have limitations in diagnosing current disease activity, as they solely provide insights into past disease activity [6].

Complexities and shortcomings of conventional diagnostic approaches

The advancements in biomedical technology, particularly regarding high-throughput methods, sensitive diagnostic platforms, and machine learning algorithms, have undermined the limitations of the conventional diagnostic approaches [7]. The advent of the keystone pathogen hypothesis has made understanding of the periodontal microbiome better [8]. It has been observed that the keystone pathogen *Pophyromonas gingivalis* has the propensity to alter the milieu of a periodontal pocket from having symbiotic microflora to a dysbiotic one [9]. The emphasis is now focused on the interaction between the dysbiotic microbiome and inflammatory mediators that cause periodontitis [10,11]. The risk factors for periodontitis include environmental, genetic, and systemic factors. Viral and fungal infections also deplete the immune response of the host, and individuals with an overreactive genetic predisposition are predisposed to severe forms of periodontitis [12]. As the disease is caused by a multitude of factors, traditional diagnostic measures fall short of assessing the disease comprehensively. The advent of biomarkers has made the diagnosis of periodontitis much easier and more specific [13].

Advantages of precision over traditional periodontal diagnosis and treatment

A precision periodontal diagnosis enables the customization of disease-prevention strategies based on an individual's unique genetic, environmental, and lifestyle factors. It facilitates the prescription of more effective drugs that are tailored to an individual's specific genetic makeup, thereby avoiding drugs with predictable negative side effects. Precision medicine reduces the time, cost, and failure rate of pharmaceutical clinical trials by identifying responders and non-responders to a particular therapy. It eliminates trial-and-error inefficiencies that inflate healthcare costs and undermine patient care. Finally, it shifts the emphasis in medicine from reaction to prevention by predicting susceptibility to disease and improving disease detection.

Biomarkers in precision periodontics 

Biomarkers are an objectively measured indicator of biological processes, pathological conditions, or therapeutic responses. It allows for the real-time monitoring of biological processes by assessing regulatory molecules or by-products synthesized and released by cells and tissues in response to genetic and epigenetic factors [14]. Periodontal samples include plaque, saliva, and gingival cervicular fluid (GCF). The classification of biomarkers is thus based on information collected from the patient’s history and the diagnostic tests that evaluate the clinical stage of diseases [15].

Predictive Markers 

These help clinicians pinpoint patients at risk of disease, enabling proactive modification of screening protocols and risk factor adjustments to maximize disease prevention efforts. Single nucleotide polymorphisms (SNPs) are the best example of predictive markers, as they can identify if the patient is genetically susceptible to disease [15]. Patients with specific immunophenotypes are considered to be more prone to impaired elimination of periodontal pathogens, resulting in excessive periodontal destruction. It has been proposed that SNPs in the IL1β, IL1RN, FcγRIIIb, VDR, and TLR4 genes may underline susceptibility to more destructive forms of periodontitis, while polymorphisms in the IL1B, IL1RN, IL6, IL10, VDR, CD14, TLR4, and MMP1 genes might be responsible for general susceptibility to chronic periodontitis [16]. A study conducted by Schulz et al. on SNPs in the IL-1 gene cluster and subgingival colonization of microorganisms observed that the genetic makeup of the IL-1 gene cluster correlated with the likelihood of *Aggregatibacter actinomycetemcomitans* colonization in subgingival areas. It was concluded that there is no evidence to state that this correlation is an independent risk indicator for periodontitis [17].

Prognostic Markers

These are assessed upon disease onset and do not require temporal variation. Commonly utilized markers are genetic. These biomarkers serve as a valuable tool in treatment planning, enabling clinicians to predict potential issues, select the most effective treatment pathway, and establish a tailored maintenance plan to ensure long-term stability and optimal patient outcomes [18]. Prognostic markers are measured when disease occurs; they do not need to change over time, and they serve to estimate disease characteristics, and the stage and grade, which are indispensable for accurate prognostics of the progression pattern and responsiveness to different treatment protocols [19]. A meta-analysis conducted by Feng et al. revealed that the IL-1A (-889C/T) polymorphism was significantly associated with an increased susceptibility to chronic periodontitis in African, European, and American populations [20].

Diagnostic Markers

This group encompasses biochemical and microbiological markers, as they can identify various important parameters related to the disease activity. To assess the patient's compliance with treatment, surrogate markers, including inflammatory, tissue response products, and bone health markers, are indicated and categorized in this group [21].

Inflammatory Biomarkers

These markers include both proinflammatory and anti-inflammatory markers. Since these markers are elevated in both gingivitis and periodontitis, they are preferably used to estimate disease activity, progression, and compliance with administered treatment. The most investigated markers in periodontology are IL-1β, IFNγ, and TNFα from the T-helper (Th)-1 sub-family; IL-6, IL-4, and IL-10 from the Th-2 sub-family; IL-17 from the Th-17 sub-family; and IL-8. In general, Th-1 and Th-17 markers are generally increased in active periodontitis and usually decrease following treatment, while Th-2 markers seem to be slightly less specific than Th-1 and Th-17 [22-25]. A disbalance between reactive oxygen species (ROS) and antioxidants is a pathological characteristic of periodontal destruction. The markers that show a specific profile and good treatment responsiveness to periodontal treatment are malondialdehyde, nitric oxide, total oxidant status, total antioxidant capacity, and 8-hydroxydeoxyguanosine measured in saliva, while GCF profiles are slightly less specific. Calprotectin, which inhibits immunoglobulin production and functions as a proinflammatory protein, offers protection against bacterial invasion of epithelial cells, particularly *P. gingivalis*, by increased expression at the site of inflammation [26,27]. Becerik et al. analyzed the levels of calprotectin, osteocalcin, and cross-linked N-terminal telopeptide (NTx) in GCF in both healthy individuals and those with various periodontal diseases. The study found that elevated calprotectin levels in GCF can be a reliable indicator of inflammation in the development of periodontal disease, while the fluctuating levels of osteocalcin and NTx in GCF may suggest abnormal bone turnover in periodontitis [28].

*Soft Tissue Biomarkers* ​​​​​​

These markers serve as tissue degradation indicators, which include matrix metalloproteinases (MMPs). Both MMP8 and MMP9 levels are increased in periodontitis. Also, both MMP13 and MMP8 have been observed to be elevated in patients with periimplantitis. Platelet-derived growth factor (PDGF) supports healing, and increased vascular endothelial growth factor (VEGF) expression in epithelial cells and endothelial cells in periodontitis-affected gingiva may be a useful marker for periodontal healing [29]. A meta-analysis by Ghassib et al. examined the use of biomarkers in peri-implant crevicular fluid (PICF) to distinguish between healthy implants, peri-implant mucositis, and peri-implantitis. They found that pro-inflammatory cytokines such as IL-1β and IL-6 in PICF can be used as adjunct tools to clinical parameters to accurately identify healthy implants from those with peri-implant mucositis or peri-implantitis [30].

Bone Turnover Markers (BTMs)

The activity of bone can be assessed with the receptor activator of nuclear kappa B (RANK) and its ligand RANKL, which are predominantly associated with bone resorption, and their antagonist osteoprotegerin (OPG) associated with bone apposition and remodeling, but their diagnostic value in assessing disease activity has not yet been established [31]. Osteonectin has a vital role in the early phase of mineralization and can serve as a sensitive marker for detecting periodontitis, with higher sensitivity compared to the N-propeptide of type I collagen. Osteopontin (OPN) is critical for bone remodeling, and it has been observed that when increased levels of OPN were detected in samples, clinically the probing pocket depth was less [32].

Histopathological Markers

Although these are not commonly utilized in routine periodontal diagnosis, they can offer valuable insights into the nature, progressive pattern, and severity of the disease. The assessment of these markers may contribute to the selection of biomarkers that are more suitable for practical use in clinical settings.

Gingival cervicular fluid as a source of biomarkers

The diagnostic potential of GCF has been extensively established, as evidenced by a huge array of biomarkers identified in its composition. This property renders GCF more advantageous for clinical use compared to other fluids. Studies have demonstrated a correlation between proteases and collagenase levels in GCF and periodontal disease-related pocket depth derangements. Barros et al. have highlighted the fact that the GCF serves as a reservoir for a multitude of biomarkers that may indicate the presence of disease, with distinctions between active and passive sites. In a review published in 2017, Kaur et al. presented an extensive analysis of host defense mediators, wherein they highlighted the role of GCF as a source for biomarker analysis. The site-specificity of GCF makes it a good source for chairside diagnostic tests, though the collection of GCF samples is laborious and technique-sensitive [33].

Saliva as a source of biomarkers

Salivary analysis offers a non-invasive alternative for diagnosing oral diseases due to its ease of sample acquisition, simpler processing, less complex composition, and greater stability compared to other sources. Saliva provides real-time information as it is continuously produced by exocrine glands. Additionally, saliva contains various biomolecules that can aid in diagnosis and prognosis, including metabolites, proteins, mRNA, DNA, enzymes, hormones, antibodies, antimicrobial constituents, and growth factors. IL-1β, MMP-8, IL-6, and MMP-9 were most frequently analyzed in saliva, followed by IL-8, TIMP-1, IL-10, and MMP-3, which were elevated in the case of periodontitis. The analysis of salivary biomarkers associated with osteoclastogenic cytokines, such as RANK, RANKL, and OPG, as well as antioxidant proteins, including urate, malondialdehyde, ascorbate, and myeloperoxidase, IL-1, IL-6, IL-8, and IL-12, were elevated in patients with peri-implantitis. The TNF levels have also been reported to be higher in patients with peri-implantitis than in healthy individuals. This highlights the lack of consensus regarding which biomarkers should be used for specific scientific targets and purposes. As a result, no clear recommendations can currently be made regarding salivary biomarkers for personalized oral healthcare. Both MMPs and ILs appear to be the most promising biomarkers, particularly in the context of periodontology [34].

Strategies to implement precision in periodontics

The emergence of advanced technologies for comprehensive biological profiling of patients has initiated the transition towards precision periodontics, although this is still in its early stages. The type of biomarker to be included in the examination depends upon what has to be examined. For microbiological tests, one can use metagenomic and meta-transcriptomic methods, along with cultomics. If the levels of cytokines have to be assessed, then the utility of proteomics is mandatory. Depending on which aspect of the disease has to be examined, the choice of biomarker is made. However, biomarker analysis is also fraught with many limitations, like false positive or false negative results, which have to be overcome by superior equipment and techniques with experienced and well-trained personnel.

Precision Dentistry and Advanced Technology

Precision dentistry has revolutionized the way clinicians work, allowing for more accurate and efficient treatment. Modern dental setups utilize digital technology, enabling real-time monitoring of patients’ vital signs and equipment performance. Radio frequency identification (RFID) tags track dental instruments, improving clinic efficiency and data collection. Digital dentistry has also transformed dental restorations, with computer-aided design and manufacturing replacing traditional methods. The 3D scanners have made uncomfortable dental-impression recording a thing of the past, while high-end microscopes enable precise procedures such as root canal therapy and tissue grafts. These advancements have significantly improved patient care and outcomes [35].

Artificial Intelligence, a Boost to Precision Dentistry

The convergence of precision dentistry and artificial intelligence (AI) yields innovative solutions for improved patient care. The AI's ability to analyze large datasets complements precision dentistry's focus on individualized care. This synergy enables improved diagnostics through AI's pattern recognition capabilities. Personalized treatment and predictive maintenance of dental equipment are possible. It enables patient engagement through AI-powered gadgets and virtual assistants and accelerated research and development of new treatments. However, several challenges must be addressed, including the security of data, compliance with regulations, education, and training of dental professionals, and cost implications, before success can be achieved [36].

Barriers to precision periodontics

**Figure 1:**
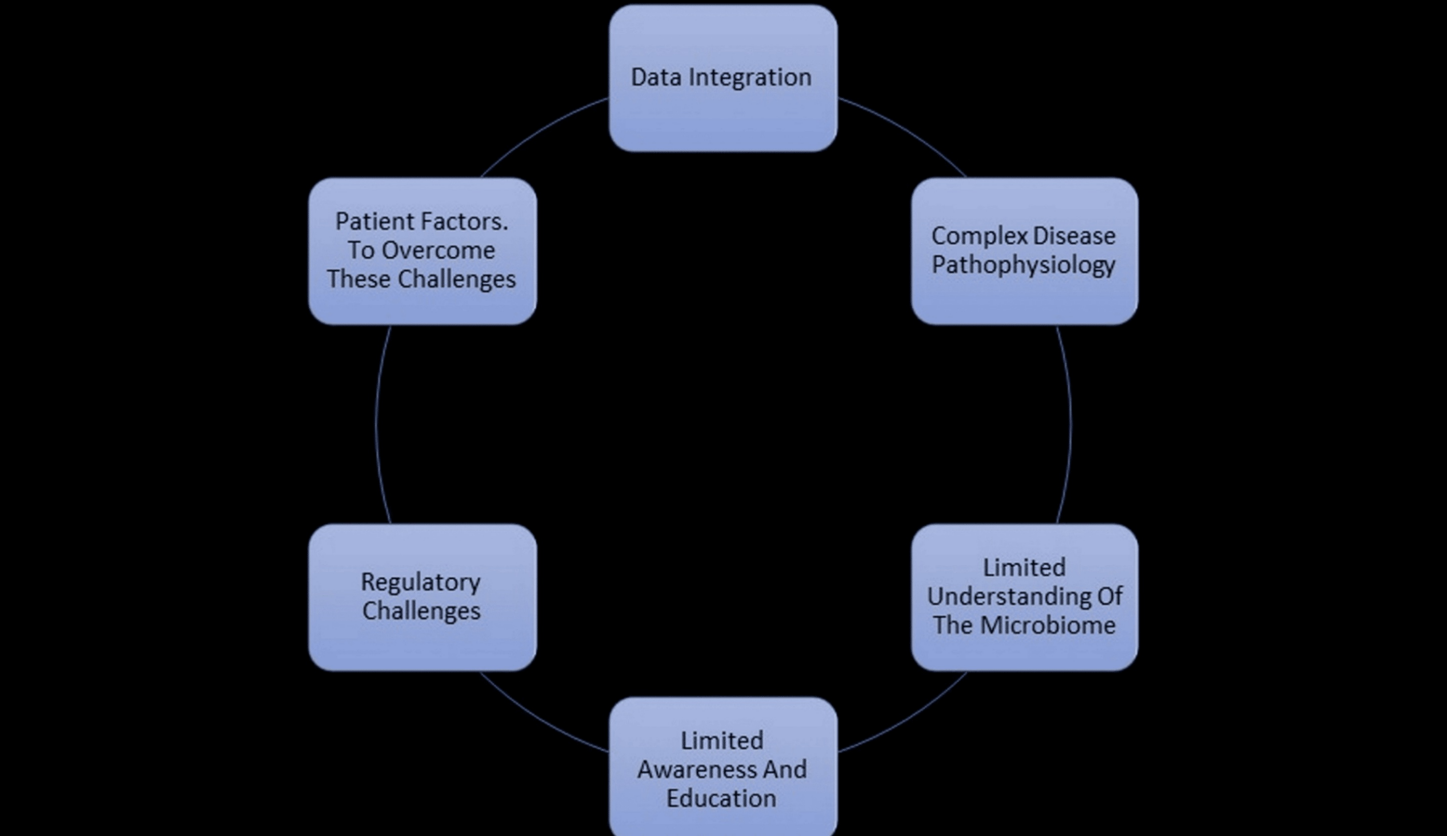
Barriers to precision periodontics This flowchart was created by the authors.

Figure 1 portrays the barriers encountered regarding precision periodontics. First is data integration, i.e., the integration and standardization of data from various sources. This can be overcome using electronic health records and precision medicine platforms and developing common data models and ontologies. Second, multiple etiologic factors make periodontal disease complex, making it challenging to identify biomarkers and diagnostic tools. Collaborative research and AI/machine learning algorithms can be helpful in this respect. Third, there is a limited understanding of the microbiome. Its composition and dynamics are still unknown, hindering antimicrobial therapies. Further research using cutting-edge technologies like 16S rRNA gene sequencing and developing novel antimicrobial therapies will be a breakthrough. Fourth, limited awareness and education are another barrier. Dental professionals need to understand advanced diagnostic tools and treatment options. Continuing education programs, workshops, and online resources may improve awareness. Fifth, rapid innovation requires adapting regulatory frameworks. Engaging with regulatory agencies to develop guidelines and standards could eliminate regulatory challenges. Sixth, patient factors such as compliance, socioeconomic status, and cultural background influence treatment outcomes. Therefore, patient-centered approaches, personalized care plans, and patient engagement should be developed [37].

Precision oral health necessitates a paradigmatic transformation, shifting from diagnostic categorization to phenotypic and risk-based stratification. This approach endeavors to classify individuals into homogenous, mutually exclusive groups based on their disease risk profiles, progression trajectories, and treatment response patterns, thereby reorienting the inquiry from 'What is this patient's disease classification?' to determining what is the patient's tendency for disease progression, tooth loss, or treatment failure.

## Conclusions

The development of precision periodontics is poised to revolutionize periodontal care, warranting a commitment to adhering to established biomarker validation protocols to accelerate their incorporation into evidence-based clinical practice. Research endeavors should focus on creating practical biomarker evaluation protocols, including point-of-care testing (POCT), which is currently in its infancy in periodontology. The absence of validated periodontal biomarkers for diagnostic purposes hampers the advances made in biomarker analysis as a tool for precision in periodontal diagnosis and treatment. Thus, the time has come to assess the most specific biomarkers related to the needs of the patient to help in making precision periodontics a reality.
